# Comparison of the Combined Obesity Indices to Predict Cardiovascular Diseases Risk Factors and Metabolic Syndrome in Northeast China

**DOI:** 10.3390/ijerph13080801

**Published:** 2016-08-09

**Authors:** Yuchun Tao, Jianxing Yu, Yuhui Tao, Hui Pang, Yang Yu, Yaqin Yu, Lina Jin

**Affiliations:** 1 Epidemiology and Biostatistics School of Public Health, NO. 1163 Xinmin Street, Jilin University, Changchun 130021, China; taoyuchun@163.com (Y.T.); yjxjlu@163.com (J.Y.); panghui15@mails.jlu.edu.cn (H.P.); yangyu15@mails.jlu.edu.cn (Yang.Y.); yuyq@jlu.edu.cn (Yaqin.Y.); 2 Department of Immunization Program, Changchun Center for Disease Control and Prevention, Changchun 130021, China; taoyuhuicfy@163.com

**Keywords:** obesity, cardiovascular diseases, metabolic syndrome, AUROC

## Abstract

*Background*: Obesity is associated with cardiovascular disease (CVD) risk factors (hypertension, dyslipidemia and diabetes) and metabolic syndrome (MetS), and it may be flawed that most studies only use one obesity index to predict these risk factors. Therefore, our study aims to compare the various combined obesity indices systematically, and to find the optimal combined obesity indices to predict CVD risk factors and MetS. *Methods*: A total of 16,766 participants aged 18–79 years old were recruited in Jilin Province in 2012. Receiver operating characteristic curve (ROC) curves and multiple logistic regressions were used to evaluate the predictive capacity of the combined obesity indices for CVD risk factors and MetS. *Results*: The adjusted area under receiver operating characteristic (AUROC) with two combined obesity indices had been improved up to 19.45%, compared with one single obesity index. In addition, body mass index (BMI) and waist circumference (WC) were the optimal combinations, where the AUROC (95% confidence interval (CI)) for hypertension, dyslipidemia, diabetes and MetS in males were 0.730 (0.718, 0.740), 0.694 (0.682, 0.706), 0.725 (0.709, 0.742) and 0.820 (0.810, 0.830), and in females were 0.790 (0.780, 0.799), 0.727 (0.717, 0.738), 0.746 (0.731, 0.761) and 0.828 (0.820, 0.837), respectively. *Conclusions*: The more abnormal obesity indices that one has the higher the risk for CVD risk factors and MetS, especially in males. In addition, the combined obesity indices have better predictions than one obesity index, where BMI and WC are the optimal combinations.

## 1. Introduction

The prevalence of obesity is developing extremely quickly worldwide nowadays, especially in China [1,2], and it is believed that obesity is closely associated with cardiovascular disease (CVD) risk factors (hypertension (HTN), dyslipidemia (DLP) and diabetes mellitus (DM)) and metabolic syndrome (MetS) in literature [3,4,5,6]. Obviously, studies focusing on this field inevitably utilize indices to denote and evaluate obesity. Thus, various obesity indices have been proposed based on different research purposes [7,8], such as body mass index (BMI), waist-hip ratio (WHR), waist-to-height ratio (WHtR), etc. In addition, the optimal obesity index varies according to the study population [9].

Some studies have indicated that BMI is a strong predictor of CVD mortality for whites [10], and is one of the most commonly used indices for obesity [11]; other studies indicated that waist circumference (WC) or WHtR might be a better predictor for CVD risk factors or MetS in Korean/Chinese and other ethnic groups [8,12,13], while Mbanya et al. pointed out that WC was the best predictor in Cameroonian [14], and WHR was viewed as the index for evaluating fat distribution [9]. Moreover, Bergman et al.’s study found that body adiposity index (BAI) was a better predictor for African-American and Mexican-American [15], while Lam et al. manifested that BAI might be unlikely to be better than BMI and was not applied to Asian [9]. Therefore, BAI is not considered in the present study.

Furthermore, a significant challenge is that body composition in Asia is markedly different in comparison with the U.S., Europe, etc. Thus, no obesity index is consistently superior to others, and the selection of obesity index depends on the study population and other factors [9]. Some studies have also indicated that one single index may be not sufficient to evaluate obesity [16], and there is probably a tendency to cluster in obesity indices. But unfortunately, less research is reported on this field, not to mention systematic studies.

In this study, various combinations of BMI, WC, WHR and WHtR are investigated to evaluate the predictive ability comprehensively for CVD risk factors and MetS. In addition, 16,766 adults aged 18–79 years old in Jilin Province have participated in our study. It turns out that the participants are at a higher risk when they have more abnormal obesity indices, which are more serious in males. In addition, the combinations of indices have better performances than a single index, where BMI and WC are the optimal pairs. In addition, Jilin is located in the central part of northeast China, with latitude 40°–46° N and longitude 121°–131° E [17], so the results may be instructive and meaningful to the studies related to obesity in northeast China.

## 2. Methods

### 2.1. Study Population

A total of 16,766 adults aged 18–79 years old were selected through multistage stratified random cluster sampling in Jilin Province in 2012. These participants had lived in Jilin Province for more than 6 months (for a detailed sampling method, see Section 1 of online Supplementary Material).

### 2.2. Ethics Statement

The ethics committee of the School of Public Health, Jilin University approved the study (project identification code: 2012-R-011), and written informed consent was obtained from all of the participants before data collection.

### 2.3. Data Measurement

Height, weight, WC, hip circumference (HC) and blood pressure were measured by trained professionals, with the participants wearing light clothing but no shoes. Blood pressure was measured using a mercury sphygmomanometer. After an overnight fast, serum lipids and fasting blood glucose (FBG) were measured before breakfast, using a MODULE P800 biochemical analysis machine (Roche Co., Ltd., Shanghai, China) and a Bai Ankang fingertip blood glucose monitor (Bayer, Leverkusen, Germany), respectively.

The various obesity indices were calculated as follows:

(1) $$\text{BMI}=\frac{\text{weight(kg)}}{{\text{height}}^{2}\text{(m)}},\;\text{WHR=}\frac{\text{WC(cm)}}{\text{HC(cm)}},\;\text{WHtR=}\frac{\text{WC(cm)}}{\text{height(cm)}}$$

### 2.4. Assessment Criteria

In the study, hypertension (HTN) was defined as resting systolic blood pressure (SBP) ≥ 140 mmHg and/or diastolic blood pressure (DBP) ≥ 90 mmHg and/or the use of antihypertensive medication in the past two weeks [18]. Dyslipidemia (DLP) was defined as using lipid-lowering drugs or having one or more of the following: triglyceride (TG) ≥ 1.7 mmol/L, total cholesterol (TC) ≥ 5.2 mmol/L, high-density lipoprotein cholesterol (HDL-C) < 1.0 mmol/L and low-density lipoprotein cholesterol (LDL-C) ≥ 3.4 mmol/L [19]. diabetes mellitus (DM) was defined as the use of hypoglycemic agents or a self-reported history of DM or FBG of 7.0 mmol/L or more [20]. MetS was defined as three or more of the following conditions clustered in one subject: (a) WC ≥ 85 cm for males or ≥80 cm for females; (b) TG ≥ 1.7 mmol/L or ongoing hypertriglyceridemia treatment; (c) HDL-C < 1.0 mmol/L for males or <1.3 mmol/L for females, or ongoing treatment; (d) SBP ≥ 130 mmHg and DBP ≥ 85 mm Hg, or ongoing antihypertensive drug therapy; and (e) FBG ≥ 5.6 mmol/L or ongoing anti-diabetic drug treatment [21,22]. The optimal cut-off values of obesity indices recommended by others studies [23,24,25] as well as the present studies were listed in Table 1, where subscript “0/1” denoted normal/abnormal for the index.

### 2.5. Statistical Analyses

The quantitative variables were expressed as means ± standard deviations (SD) and compared using a Student’s *t*-test. The categorical variables were expressed as counts, percentages and compared using a Rao–Scott-*χ*^2^ test. Area under receiver operating characteristic (AUROC) analyses were used to evaluate the predictive ability. Logistic regression models were used to calculate the odds ratios (ORs) and to evaluate the combined obesity indices. All statistical analyses were performed using the IBM SPSS 20.0 (SPSS Inc., New York, NY, USA) and the R version 3.3.1 (University of Auckland, Oakland, New Zealand). Statistical significance was set at *p*-value < 0.05.

## 3. Results

The basic characteristics of the participants are given in Table 2. The percentages of BMI_1_, WHR_1_, HTN, DLP, DM and MetS in males were significantly higher than those in females (*p* < 0.05), while WC_1_ did not differ statistically significantly by gender (*p* > 0.05). 

**Figure 1:**
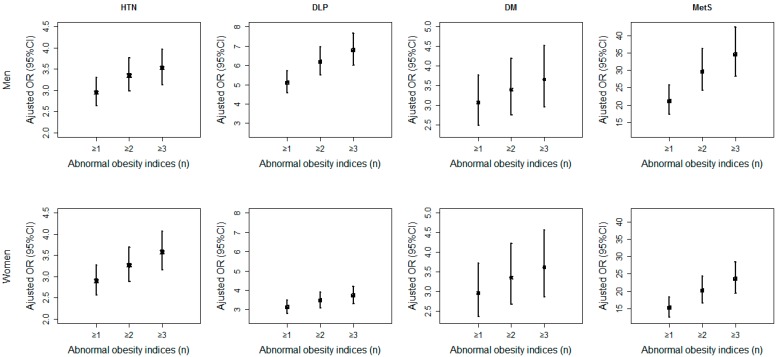
The clustering effects of the obesity indices on cardiovascular disease (CVD) risk factors and metabolic syndrome (MetS).

Figure 1 shows that the adjusted ORs were increasing obviously with the clustering of the abnormal obesity indices (where ≥1, ≥2 and ≥3 refer to the sum of number of abnormal BMI, WC, WHtR and WHR) compared with the normal obesity indices, that is, the person with more abnormal obesity indices was at a higher risk for CVD risk factors and MetS. Meanwhile, almost all the adjusted ORs in males (1st row of Figure 1) were larger than those in females (second row of Figure 1), especially for DLP and MetS (see details in online Supplementary Table S1).

**Figure 2:**
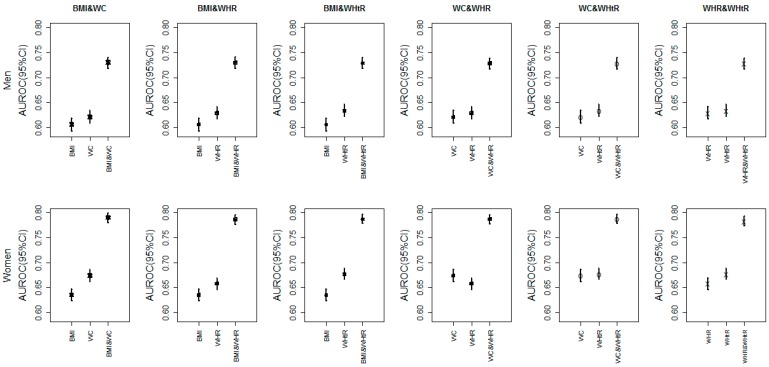
The adjusted area under receiver operating characteristic (AUROC) of a single index vs. various combinations of two indices for hypertension (HTN).

Since we had the evidence that the clustering of obesity indices would increase the risk for CVD risk factors and MetS, we now investigated how much it could be improved with combinations of obesity indices using AUROC. Figure 2 shows the adjusted AUROC of a single index vs. various combinations of two indices for HTN, respectively (DLP, DM and MetS were in the online supplement Figures S1–S3). And it was obvious that the predictive capacity for HTN with two obesity indices was much better than that with one single index. For example, the adjusted AUROC of BMI and WHR for HTN in females were 0.635 and 0.658, respectively, but the adjusted AUROC of the combination BMI and WHR were 0.786 (online Supplementary Tables S2 and S3), which had increased by at least 0.128 (0.128/0.658 = 19.45%). Besides, the rise in AUROC for HTN and DM (at least 12.37%–19.45%) was larger than DLP and MetS (at least 2.27%–12.29%), and generally the rise in AUROC for females was larger than that for males. In addition, we investigated how the combinations of two obesity indices behaved in predicting CVD risk factors and MetS in different age groups as well (online Supplementary Table S4). It was shown that the younger age groups had better performances (larger AUROC). However, combinations with more than two obesity indices were not considered in our studies, due to the potential collinearity.

**Figure 3:**
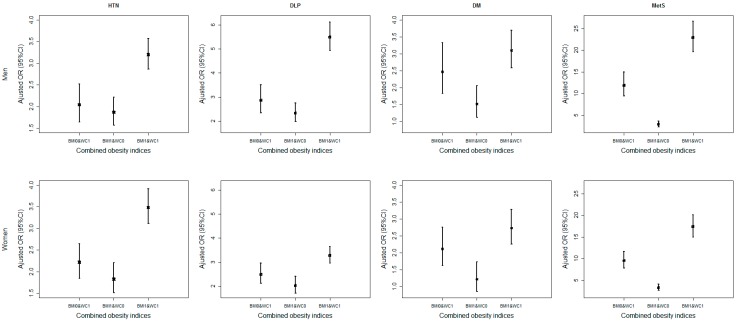
The adjusted odds ratios (ORs) of the combination body mass index (BMI) and waist circumference (WC) for CVD risk factors and MetS.

Finally, the effects of BMI and WC are shown in Figure 3 (and other combinations are in online Supplementary Tables S5–S7). Four categories were reassigned for each combination, from no abnormal to two abnormal obesity indices (i.e., 00, 01, 10 and 11). Generally, the adjusted ORs for the two abnormal obesity indices were much higher than those with any single abnormal obesity index, especially for MetS. Besides, the adjusted ORs for BMI_0_ and WC_1_ were a little higher than BMI_1_ and WC_0_, that is, abnormal WC might have a higher risk than abnormal BMI.

## 4. Discussion

The prevalences of HTN, DLP, DM and MetS in our study were 37.27%, 39.76%, 10.07% and 33.1%, respectively, which were much higher than those in other studies [19,26,27]. The reason might be the different study populations, different survey times, different threshold criteria, and different diet patterns. Firstly, the threshold criteria for MetS, WC, etc. might be different in different studies [21,28,29,30]. Secondly, the climate of residence to survey population usually requires a special diet such as eating more animal fat, more salt and fewer fresh vegetables, while the diet patterns are believed to be associated with different degrees of MetS prevalence, and lower salt intake can reduce MetS [31,32,33]. Finally, the population participates in fewer outdoor activities (exercises), especially during the cold winter months [34]. 

Meanwhile, a number of studies had demonstrated that obesity was associated with CVD risk factors and MetS [3,35], and various obesity indices were used in literature [36,37] to describe obesity. Furthermore, some studies also indicated that one single index might be not sufficient to evaluate obesity [16], but, unfortunately, less research was reported on this field, not to mention systematic studies.

In our study, we investigated four obesity indices, BMI, WC, WHR and WHtR, together with their clustering in predicting CVD risk factors and MetS. It turned out that the participants were at an increasing risk with the of abnormal obesity indices that one had. Generally, the adjusted ORs in males were higher than those in females, especially for DLP and MetS. One possible reason was that the prevalence of abdominal obesity in males might be more common than that in females, and the other possible reason was that females tended to control their weight in contrast with males [38,39]. Therefore, more effective attention and intervention should be paid to obesity, especially abdominal obesity in males.

Furthermore, using the combination of two indices could improve the AUROC on different levels, and the rise was up to 19.45%. Meanwhile, a number of previous studies also suggested that the combination of two indices had a better predictive capacity than either alone [40,41]. The reason might be that the combinations of different indices incorporated the information both in the amount of fat and in the distribution of fat. Similarly, the rise of AUROC for HTN and DM were larger than DLP and MetS, which might be due to the fact that DLP and MetS were primarily influenced by abdominal obesity; while HTN and DM also depended on the amount of fat to some extent. In addition, BMI and WC were the preferable combinations in general, which was in agreement with Zhu et al.’s and Ricketts et al.*’*s study [40,42]. Besides, the AUROC of younger age groups was the largest in most cases, which implied that obesity might have much more impact on young people.

Finally, we demonstrated the adjusted ORs of each combined obesity indices for CVD risk factors and MetS. There was no surprise to find that the participant with two abnormal indices (11) gained the highest risk than other cases (00, 01 and 10) for all combinations and risk factors. Moreover, it seemed that WC had played an important role in evaluating obesity for CVD risk factors and MetS. For example, the adjusted ORs for BMI_0_ and WC_1_ were a little higher than BMI_1_ and WC_0_, that is, abnormal WC might have a higher risk than abnormal BMI, and similar findings were also obtained in other combinations with WC. Therefore, it was implied that the distribution of fat had a greater influence on CVD risk factors and MetS than the amount of fat.

Some limitations of our study should be noted here. Firstly, the definition of MetS had an overlap with WC, which might lead to overestimations of adjusted AUROC and ORs for MetS. Secondly, age was adjusted in our studies; however, other confounders that might have impact on CVD risk factors and MetS [42,43,44,45,46,47,48], such as physical activity or fitness, smoking, etc., were not under our consideration. Finally, the results in our study were attained from Jilin province, which might limit our ability to generalize the results to the rest of China. 

## 5. Conclusions

The adjusted ORs for CVD risk factors and MetS increase with the amount of abnormal obesity indices, especially in males. The AUROC with two indices has been improved up to 19.45% compared with that of one single index, where the rise of AUROC in females is larger than that in males, and the optimal combinations are BMI and WC. In addition, abdominal obesity has more impact on most CVD risk factors and MetS, and the risk of abdominal obesity for CVD risk factors and MetS in males is larger than that in females, which imply that more effective attention and intervention should be paid to males.

## Figures and Tables

**Table 1:** Optimal cut off values of obesity indices and corresponding abbreviations.

Index	Cut-off	Category	Abbreviation
BMI	24	BMI ≤ 24 kg/m^2^	BMI_0_
		BMI > 24 kg/m^2^	BMI_1_
WC	85 (male)	WC ≤ 85(male) or WC ≤ 80 (female)	WC_0_
	80 (female)	WC > 85(male) or WC > 80 (female)	WC_1_
WHR	0.88 (male)	WHR ≤ 0.88(male) or WHR ≤ 0.85 (female)	WHR_0_
	0.85 (female)	WHR > 0.88(male) or WHR > 0.85 (female)	WHR_1_
WHtR	0.5	WHtR ≤ 0.5	WHtR_0_
		WHtR > 0.5	WHtR_1_

**Table 2:** Descriptive characteristics of participants by gender.

Variable	All	Male	Female	*t/χ*^2^	*p*-Value
	(*n* = 16,766)	(*n* = 7697)	(*n* = 9069)		
Age (year)	47.80 ± 13.18	47.00 ± 13.74	48.47 ± 12.66	−7.20	<0.001
BMI_1_	8467 (50.50%)	3982 (51.73%)	4485 (49.45%)	8.66	0.003
WC_1_	8077 (48.17%)	3651 (47.43%)	4426 (48.80%)	3.13	0.077
WHR_1_	8550 (50.00%)	4152 (53.94%)	4398 (48.49%)	49.45	<0.001
WHtR_1_	8826 (52.64%)	3856 (50.10%)	4970 (54.80%)	36.96	<0.001
HTN	6249 (37.27%)	3162 (41.08%)	3087 (34.04%)	88.31	<0.001
DLP	6679 (39.76%)	3410 (44.30%)	3269 (36.05%)	118.44	<0.001
DM	1688 (10.07%)	820 (10.65%)	868 (9.57%)	5.39	0.020
MetS	5535 (33.01%)	2638 (34.27%)	2897 (31.94%)	10.21	0.001

## Supplementary Materials

The following are available online at www.mdpi.com/1660-4601/13/8/801/s1, Figure S1: the adjusted AUROC of a single index and various combinations of two indices for dyslipidemia, Figure S2: the adjusted AUROC of a single index and various combinations of two indices for diabetes, Figure S3: the adjusted AUROC of a single index and various combinations of two indices for MetS, Table S1: the clustering effects of the obesity indices on CVD risk factors and MetS, Table S2: AUROC of the various obesity indices for CVD risk factors and MetS, Table S3: AUROC of the clustering of the various obesity indices for CVD risk factors and MetS, Table S4: AUROC of the two obesity indices combinations to predict CVD risk factors and MetS in different age groups, Table S5: the adjusted ORs of the combined obesity indices for CVD risk factors and MetS, Table S6: the adjusted ORs for the combined obesity indices with CVD risk factors and MetS, Table S7: the adjusted ORs for the combined obesity indices with CVD risk factors and MetS.

## Abbreviations

The following abbreviations are used in this manuscript:

WC	waist circumference
BMI	body mass index
WHR	waist-hip ratio
WHtR	waist-to-height ratio
HC	hip circumference
BAI	body adiposity index

The laboratory biochemical indicators:

SBP	systolic blood pressure
DBP	diastolic blood pressure
TC	total cholesterol
TG	triglyceride
LDL-C	low-density lipoprotein cholesterol
HDL-C	high-density lipoprotein cholesterol
FBG	fasting blood glucose

Other indicators:

CVD	cardiovascular disease
HTN	hypertension
DLP	dyslipidemia
DM	diabetes mellitus
MetS	metabolic syndrome
OR	odds ratio
CI	confidence interval
ROC	receiver operating characteristic curve
AUROC	area under ROC
