# Supplementary material for: Comparison of the Combined Obesity Indices to Predict Cardiovascular Diseases Risk Factors and Metabolic Syndrome in Northeast China
Source: Int J Environ Res Public Health. 2016 Aug 9;13(8):801. doi: 10.3390/ijerph13080801 (PMC4997487; doi:10.3390/ijerph13080801)
Supplement: Supplementary file 1 [file ijerph-13-00801-s001.pdf]

Supplementary Material: Comparison of the Combined Obesity Indices to Predict Cardiovascular Diseases Risk Factors and Metabolic Syndrome in Northeast China

Yuchun Tao, Jianxing Yu, Yuhui Tao, Hui Pang, Yang Yu, Yaqin Yu and Lina Jin

1. Sampling Method

Five-stage stratified random cluster sampling was used to select the samples under study. In the first stage, 32 districts/counties were identified in proportion to population, geographic location and ethnicity, from nine cities (Changchun, Jilin, Siping, Liaoyuan, Tonghua, Baishan, Songyuan, Baicheng and Yanbian). At the second stage, three or four towns (depending on the size of the district) were selected by stratified random sampling to guarantee the representativeness of each sample. In the third stage, three neighborhood committees were chosen by stratified random sampling from each of the towns previously selected. In the fourth stage, one village from each chosen neighborhood committee was selected by simple random sampling. In the final stage, cluster random sampling was used to identify individuals aged 18 to 79 years old from each of the villages selected for the study.

2. Data Measurement

Anthropometric measurements including height, weight, waist circumference (WC), hip circumference (HC), blood pressure, serum lipids and fasting blood sugar were taken. During the interview, weight, height, WC and HC were determined though standardized protocol and measured in light indoor light clothing without shoes. WC was measured midway between the inferior margin of the last rib and the crest of the ileum in a horizontal plan. HC was measured at the maximal protrusion of the buttocks. Weight was measured to the nearest 0.1 kg, and height, WC and HC were measured to the nearest 0.1 cm.

A mercury sphygmomanometer was used to measure the blood pressure in the sitting position after a 10-min rest period. The appearance of the first sound was used to define systolic blood pressure (SBP) and the disappearance of sound was used to define diastolic blood pressure (DBP). Two readings each of SBP and DBP were recorded, and the average of each measurement was used for data analysis. If the first two measurements differed by more than 5 mmHg, additional readings were taken.

Blood samples were obtained from the antecubital vein into anticoagulant tubes containing EDTA in the morning after an overnight fasting period. All of the collected samples were transported on dry ice at prearranged intervals to the central laboratory. Serum lipids including TC, TG, HLD-C and LDL-C, which were measured by a MODULE P800 biochemical analyses machine.

Fasting plasma glucose (FPG) levels were measured using the Bayer Bai Ankang fingertip blood glucose monitor machine by taking a small drop of blood from a finger onto a strip of paper in the morning after participants fasted for 10 or more hours overnight.

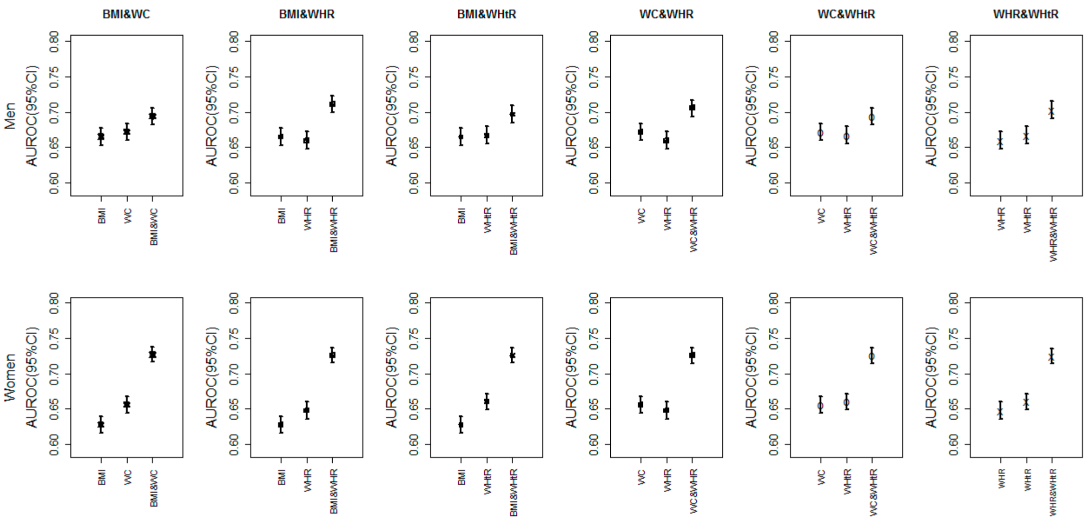

Figure S1. The adjusted AUROC of a single index and various combinations of two indices for dyslipidemia.

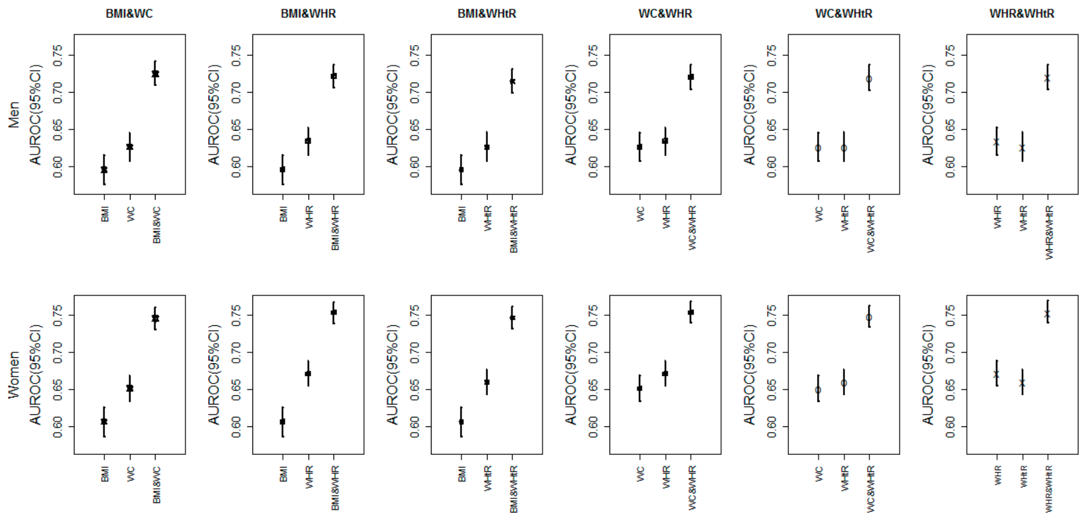

Figure S2. The adjusted AUROC of a single index and various combinations of two indices for diabetes.

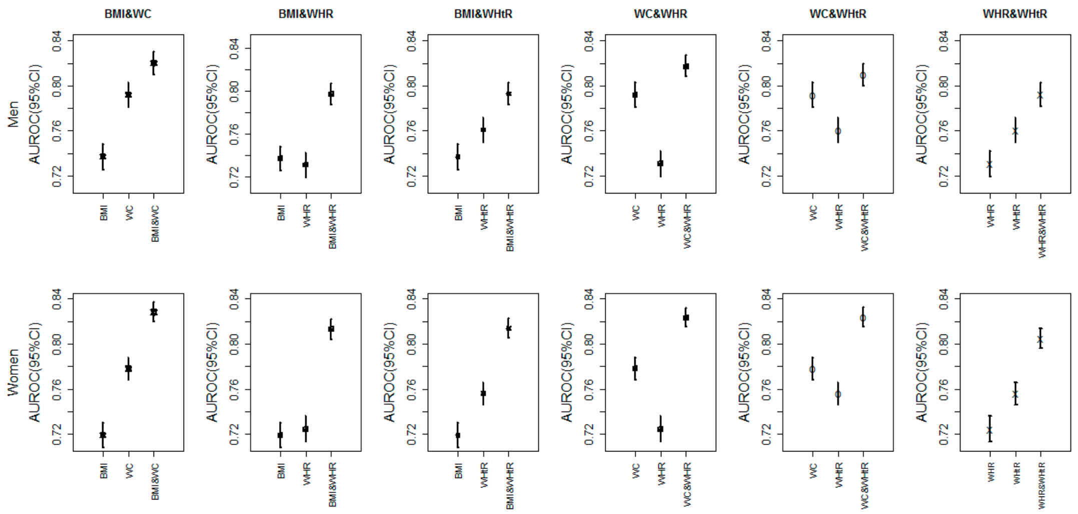

Figure S3. The adjusted AUROC of a single index and various combinations of two indices for MetS.

Table S1. The clustering effects of the obesity indices on CVD risk factors and MetS.

Risk Factors	The Adjusted ORs for Various Obesity Indices Clusters		
	≥1	≥2	≥3
**Male**			
Hypertension	2.95 (2.64,3.30)	3.35 (2.99,3.76)	3.53 (3.13,3.97)
Dyslipidemia	5.10 (4.56,5.71)	6.19 (5.50,6.96)	6.78 (6.01,7.65)
Diabetes	3.07 (2.49,3.77)	3.40 (2.76,4.20)	3.66 (2.96,4.52)
MetS	21.14 (17.31,25.82)	29.58 (24.16,36.23)	34.57 (28.18,42.40)
**Female**			
Hypertension	2.90 (2.57,3.28)	3.27 (2.89,3.70)	3.58 (3.16,4.07)
Dyslipidemia	3.12 (2.79,3.50)	3.46 (3.08,3.89)	3.72 (3.30,4.19)
Diabetes	2.96 (2.36,3.72)	3.36 (2.67,4.22)	3.62 (2.87,4.56)
MetS	15.20 (12.60,18.34)	20.15 (16.67,24.37)	23.54 (19.43,28.51)

ORs were adjusted for age.

Table S2. AUROC of the various obesity indices for CVD risk factors and MetS.

Risk Factors	BMI	WC	WHR	WHtR
	AUROC(95% CI)	AUROC(95% CI)	AUROC(95% CI)	AUROC(95% CI)
**Male**				
Hypertension	0.606 (0.593,0.619)	0.621 (0.608,0.634)	0.629 (0.617,0.642)	**0.634 (0.622,0.647)**
Dyslipidemia	0.665 (0.653,0.677)	**0.672 (0.660,0.684)**	0.660 (0.648,0.672)	0.667 (0.655,0.680)
Diabetes	0.595 (0.575,0.615)	0.626 (0.606,0.645)	**0.634 (0.615,0.652)**	0.626 (0.607,0.646)
MetS	0.737 (0.725,0.748)	**0.792 (0.781,0.803)**	0.731 (0.719,0.742)	0.761 (0.750,0.772)
**Female**				
Hypertension	0.635 (0.623,0.647)	0.674 (0.662,0.686)	0.658 (0.646,0.669)	**0.677 (0.666,0.689)**
Dyslipidemia	0.628 (0.616,0.640)	0.656 (0.644,0.668)	0.648 (0.636,0.660)	**0.661 (0.649,0.672)**
Diabetes	0.606 (0.586,0.625)	0.651 (0.633,0.669)	**0.671 (0.654,0.689)**	0.660 (0.643,0.677)
MetS	0.719 (0.708,0.730)	**0.778 (0.768,0.788)**	0.724 (0.713,0.736)	0.756 (0.746,0.766)

AUROC were adjusted for age.

Table S3. AUROC of the clustering of the various obesity indices for CVD risk factors and MetS.

Risk Factors	BMI&WC	BMI&WHR	BMI&WHtR	WC&WHR	WC&WHtR	WHR&WHtR
	AUROC(95% CI)	AUROC(95% CI)	AUROC(95% CI)	AUROC(95% CI)	AUROC(95% CI)	AUROC(95% CI)
**Male**						
Hypertension	**0.730 (0.718,0.740)**	**0.730 (0.718,0.741)**	0.729 (0.718,0.740)	0.728 (0.717,0.739)	0.728 (0.717,0.740)	0.728 (0.717,0.739)
Dyslipidemia	0.694 (0.682,0.706)	**0.711 (0.700,0.723)**	0.697 (0.685,0.709)	0.706 (0.694,0.717)	0.694 (0.682,0.706)	0.703 (0.691,0.715)
Diabetes	**0.725 (0.709,0.742)**	0.722 (0.706,0.738)	0.715 (0.699,0.732)	0.721 (0.704,0.737)	0.720 (0.703,0.737)	0.721 (0.704,0.737)
MetS	**0.820 (0.810,0.830)**	0.797 (0.787,0.807)	0.793 (0.783,0.803)	0.817 (0.808,0.827)	0.810 (0.800,0.820)	0.793 (0.782,0.803)
**Female**						
Hypertension	**0.790 (0.780,0.799)**	0.786 (0.776,0.795)	0.787 (0.778,0.797)	0.787 (0.777,0.796)	0.787 (0.778,0.797)	0.783 (0.774,0.793)
Dyslipidemia	**0.727 (0.717,0.738)**	0.726 (0.716,0.737)	0.726 (0.716,0.737)	0.726 (0.715,0.736)	0.726 (0.715,0.736)	0.725 (0.714,0.735)
Diabetes	0.746 (0.731,0.761)	**0.754 (0.739,0.768)**	0.747 (0.732,0.762)	**0.754 (0.740,0.769)**	0.749 (0.734,0.764)	**0.754 (0.740,0.770)**
MetS	**0.828 (0.820,0.837)**	0.813 (0.804,0.822)	0.814 (0.805,0.823)	0.823 (0.815,0.832)	0.824 (0.815,0.833)	0.805 (0.796,0.814)

AUROC were adjusted for age.

Table S4. AUROC of the two obesity indices combinations to predict CVD risk factors and MetS in different age groups.

Risk Factors	AUROC (95% CI) in Males			AUROC (95% CI) in Females		
	18–44	45–64	65–79	18–44	45–64	65–79
**Hypertension**						
BMI&WC	0.668 (0.647,0.689)	0.621 (0.603,0.639)	0.612 (0.571,0.652)	0.705 (0.678,0.732)	0.636 (0.620,0.652)	0.597 (0.557,0.636)
BMI&WHR	0.676 (0.655,0.697)	0.623 (0.605,0.642)	0.628 (0.588,0.669)	0.690 (0.663,0.717)	0.633 (0.617,0.649)	0.575 (0.536,0.614)
BMI&WHtR	0.667 (0.645,0.688)	0.622 (0.604,0.640)	0.628 (0.588,0.669)	0.700 (0.673,0.727)	0.633 (0.617,0.649)	0.587 (0.548,0.627)
WC&WHR	0.671 (0.65,0.692)	0.623 (0.605,0.641)	0.614 (0.573,0.654)	0.682 (0.654,0.711)	0.635 (0.619,0.651)	0.589 (0.550,0.629)
WC&WHtR	0.662 (0.641,0.684)	0.621 (0.603,0.639)	0.615 (0.574,0.655)	0.685 (0.657,0.714)	0.632 (0.616,0.648)	0.595 (0.556,0.634)
WHR&WHtR	0.668 (0.647,0.690)	0.624 (0.606,0.643)	0.624 (0.584,0.665)	0.679 (0.651,0.708)	0.629 (0.613,0.645)	0.558 (0.518,0.598)
**Dyslipidemia**						
BMI&WC	0.718 (0.700,0.736)	0.683 (0.666,0.700)	0.645 (0.606,0.685)	0.659 (0.635,0.682)	0.635 (0.619,0.650)	0.588 (0.551,0.625)
BMI&WHR	0.730 (0.712,0.748)	0.687 (0.670,0.704)	0.650 (0.611,0.690)	0.665 (0.642,0.688)	0.638 (0.622,0.653)	0.587 (0.551,0.624)
BMI&WHtR	0.715 (0.697,0.734)	0.675 (0.658,0.693)	0.648 (0.609,0.687)	0.659 (0.636,0.683)	0.633 (0.617,0.649)	0.585 (0.548,0.622)
WC&WHR	0.721 (0.703,0.739)	0.685 (0.668,0.702)	0.648 (0.610,0.687)	0.642 (0.618,0.667)	0.639 (0.623,0.655)	0.592 (0.555,0.628)
WC&WHtR	0.707 (0.688,0.725)	0.673 (0.656,0.691)	0.643 (0.604,0.683)	0.638 (0.613,0.662)	0.634 (0.619,0.650)	0.589 (0.552,0.626)
WHR&WHtR	0.716 (0.697,0.734)	0.677 (0.660,0.695)	0.643 (0.604,0.681)	0.646 (0.622,0.670)	0.637 (0.621,0.653)	0.579 (0.542,0.616)
**Diabetes**						
BMI&WC	0.659 (0.615,0.704)	0.618 (0.594,0.643)	0.620 (0.567,0.673)	0.680 (0.620,0.740)	0.604 (0.581,0.627)	0.575 (0.532,0.618)

BMI&WHR	0.675 (0.632,0.718)	0.626 (0.603,0.650)	0.608 (0.556,0.660)	0.702 (0.640,0.763)	0.634 (0.612,0.656)	0.568 (0.524,0.612)
BMI&WHtR	0.659 (0.615,0.704)	0.612 (0.588,0.636)	0.601 (0.549,0.654)	0.678 (0.617,0.739)	0.603 (0.581,0.626)	0.585 (0.542,0.627)
WC&WHR	0.674 (0.630,0.718)	0.634 (0.610,0.658)	0.609 (0.558,0.661)	0.693 (0.631,0.755)	0.638 (0.615,0.660)	0.559 (0.516,0.603)
WC&WHtR	0.663 (0.619,0.707)	0.621 (0.597,0.646)	0.603 (0.550,0.655)	0.675 (0.615,0.736)	0.614 (0.591,0.636)	0.571 (0.528,0.613)
WHR&WHtR	0.673 (0.628,0.717)	0.630 (0.606,0.654)	0.587 (0.536,0.639)	0.697 (0.636,0.757)	0.637 (0.615,0.659)	0.559 (0.516,0.602)
**MetS**						
BMI&WC	0.832 (0.818,0.847)	0.799 (0.784,0.813)	0.778 (0.743,0.813)	0.842 (0.824,0.859)	0.760 (0.746,0.774)	0.711 (0.678,0.744)
BMI&WHR	0.823 (0.808,0.838)	0.770 (0.754,0.785)	0.764 (0.729,0.799)	0.810 (0.791,0.828)	0.745 (0.731,0.759)	0.696 (0.663,0.730)
BMI&WHtR	0.813 (0.797,0.829)	0.769 (0.754,0.785)	0.763 (0.728,0.798)	0.824 (0.806,0.843)	0.740 (0.725,0.754)	0.695 (0.662,0.729)
WC&WHR	0.837 (0.822,0.852)	0.801 (0.787,0.816)	0.776 (0.743,0.809)	0.820 (0.80,0.840)	0.760 (0.747,0.774)	0.700 (0.666,0.733)
WC&WHtR	0.821 (0.805,0.836)	0.791 (0.776,0.806)	0.768 (0.733,0.803)	0.821 (0.801,0.840)	0.751 (0.737,0.765)	0.694 (0.660,0.728)
WHR&WHtR	0.814 (0.798,0.830)	0.772 (0.757,0.787)	0.744 (0.710,0.779)	0.807 (0.787,0.828)	0.739 (0.724,0.753)	0.654 (0.619,0.689)

Table S5. The adjusted ORs of the combined obesity indices for CVD risk factors and MetS.

Risk Factors	Adjusted ORs (95% CI) for BMI&WC				Adjusted ORs (95% CI) for BMI&WHR			
	BMI_0&WC_0	BMI_0&WC_1	BMI_1&WC_0	BMI_1&WC_1	BMI_0&WHR_0	BMI_0&WHR_1	BMI_1&WHR_0	BMI_1&WHR_1
**Male**								
Hypertension	1	2.04 (1.65,2.52)	1.87 (1.57,2.22)	3.20 (2.87,3.57)	1	1.86 (1.59,2.18)	2.14 (1.81,2.55)	3.55 (3.16,4.00)
Dyslipidemia	1	2.86 (2.34,3.51)	2.33 (1.98,2.75)	5.49 (4.93,6.11)	1	2.89 (2.47,3.38)	2.97 (2.52,3.49)	6.86 (6.09,7.74)
Diabetes	1	2.47 (1.83,3.33)	1.52 (1.12,2.06)	3.10 (2.59,3.70)	1	2.38 (1.84,3.10)	1.87 (1.36,2.57)	3.73 (3.03,4.61)
MetS	1	11.89 (9.45,14.96)	2.95 (2.35,3.70)	22.93 (19.70,26.70)	1	6.68 (5.38,8.29)	7.53 (6.04,9.38)	27.51 (22.92,33.02)
**Female**								
Hypertension	1	2.22 (1.85,2.65)	1.83 (1.51,2.21)	3.48 (3.11,3.91))	1	1.62 (1.38,1.91)	2.36 (2.01,2.76)	3.43 (3.02,3.88)
Dyslipidemia	1	2.49 (2.11,2.95)	2.03 (1.71,2.41)	3.28 (2.95,3.65)	1	2.05 (1.76,2.39)	2.25 (1.94,2.60)	3.61 (3.21,4.06)
Diabetes	1	2.12 (1.62,2.76)	1.22 (0.85,1.73)	2.73 (2.26,3.28)	1	2.52 (1.94,3.27)	1.42 (1.04,1.95)	3.64 (2.93,4.52)
MetS	1	9.56 (7.85,11.64)	3.34 (2.67,4.17)	17.40 (15.00,20.18)	1	5.15 (4.26,6.24)	6.72 (5.58,8.10)	17.29 (14.71,20.33)

All abbreviations of each category are in Table 1. ORs were adjusted for age.

Table S6. The adjusted ORs for the combined obesity indices with CVD risk factors and MetS.

Risk Factors	Adjusted ORs (95% CI) of WC&WHR				Adjusted ORs (95% CI) for BMI&WHtR			
	WC0&WHR0	WC0&WHR1	WC1&WHR0	WC1&WHR1	BMI0&WHtR0	BMI0&WHtR1	BMI1&WHtR0	BMI1&WHtR1
**Males**								
Hypertension	1	1.68 (1.43,1.97)	2.20 (1.78,2.73)	3.19 (2.85,3.57)	1	2.24 (1.84,2.74)	1.98 (1.64,2.39)	3.19 (2.86,3.56)
Dyslipidemia	1	2.58 (2.20,3.01)	3.42 (2.80,4.19)	5.91 (5.28,6.61)	1	2.68 (2.21,3.26)	2.43,2.04,2.90)	5.31 (4.77,5.91)
Diabetes	1	1.80 (1.37,2.36)	1.85 (1.26,2.72)	3.55 (2.92,4.30)	1	2.13 (1.60,2.85)	1.67 (1.19,2.34)	2.95 (2.46,3.54)
MetS	1	3.51 (2.82,4.38)	14.74 (11.66,18.64)	26.48 (22.46,31.23)	1	7.28 (5.82,9.10)	4.11 (3.30,5.13)	17.95 (15.50,20.79)
**Females**								
Hypertension	1	1.24 (1.02,1.50)	2.67 (2.24,3.19)	3.09 (2.75,3.48)	1	1.88 (1.59,2.22)	1.74 (1.38,2.19)	3.43 (3.05,3.86)
Dyslipidemia	1	1.60 (1.34,1.91)	2.31 (1.95,2.72)	3.24 (2.90,3.61)	1	2.31 (1.98,2.71)	2.01 (1.64,2.46)	3.39 (3.03,3.79)
Diabetes	1	2.26 (1.67,3.04)	1.69 (1.21,2.37)	3.62 (2.94,4.45)	1	2.33 (1.79,3.02)	0.83 (0.48,1.43)	3.08 (2.51,3.78)
MetS	1	2.78 (2.21,3.48)	11.58 (9.56,14.03)	16.57 (14.25,19.28)	1	6.84 (5.62,8.32)	3.80 (2.93,4.94)	16.70 (14.26,19.57)

ORs were adjusted for age.

Table S7. The adjusted ORs for the combined obesity indices with CVD risk factors and MetS.

Risk Factors	Adjusted ORs (95% CI) for WC&WHtR				Adjusted ORs (95% CI) for WHR&WHtR			
	WC0&WHtR0	WC1&WHtR0	WC0&WHtR1	WC1&WHtR1	WHR0&WHtR0	WHR1&WHtR0	WHR0&WHtR1	WHR1&WHtR1
**Males**								
Hypertension	1	2.00 (1.54,2.62)	1.99 (1.62,2.44)	3.00 (2.70,3.33)	1	1.58 (1.33,1.88)	2.14 ((1.74,2.63)	3.16 (2.83,3.53)
Dyslipidemia	1	3.27 (2.55,4.20)	2.50 (2.05,3.05)	4.96 (4.47,5.50)	1	2.52 (2.14,2.98)	2.96 (2.43,3.61)	5.67 (5.07,6.35)
Diabetes	1	2.32 (1.52,3.52)	1.53 (1.10,2.13)	3.01 (2.53,3.57)	1	1.77 (1.32,2.37)	1.39 (0.94,2.05)	3.27 (2.70,3.96)
MetS	1	12.35 (9.44,16.15)	3.03 (2.3,3.91)	20.04 (17.38,23.10)	1	3.92 (3.16,4.86)	7.99 (6.36,10.03)	19.47 (16.68,22.72)
**Females**								
Hypertension	1	2.01 (1.40,2.88)	1.57 (1.29,1.89)	3.23 (2.89,3.62)	1	1.24 (0.97,1.58)	2.44 (2.07,2.88)	3.07 (2.72,3.46)
Dyslipidemia	1	1.85 (1.32,2.59)	1.81 (1.52,2.16)	3.17 (2.85,3.52)	1	1.53 (1.23,1.91)	2.27 (1.94,2.64)	3.31 (2.96,3.71)
Diabetes	1	1.15 (0.53,2.52)	2.02 (1.49,2.74)	3.18 (2.61,3.89)	1	2.29 (1.55,3.38)	2.03 (1.49,2.77)	4.01 (3.21,5.02)
MetS	1	9.35 (6.67,13.11)	3.25 (2.59,4.08)	16.42 (14.15,19.06)	1	2.80 (2.12,3.70)	8.07 (6.69,9.74)	15.35 (13.12,17.95)

ORs were adjusted for age.

© 2016 by the authors; licensee MDPI, Basel, Switzerland. This article is an open access article distributed under the terms and conditions of the Creative Commons by Attribution (CC-BY) license (http://creativecommons.org/licenses/by/4.0/).
